# Editorial: Microbiological Safety of Foods

**DOI:** 10.3390/foods10010053

**Published:** 2020-12-28

**Authors:** Pasquale Russo, Vittorio Capozzi

**Affiliations:** 1 Department of Agriculture Food Natural Science Engineering, University of Foggia, Via Napoli 25, 71122 Foggia, Italy; pasquale.russo@unifg.it; 2 Institute of Sciences of Food Production, National Research Council of Italy (CNR), c/o CS-DAT, Via Michele Protano, 71121 Foggia, Italy

The management of food safety represents a global and transdisciplinary issue of great relevance for human health and crucial economic sectors [1,2]. For this reason, the scientific advances and innovations in the field of food safety management are essential activities for enterprises, consumers, governments, public and private agencies, and scientists, deserving of continuous efforts [3]. The classic food safety framework encompasses contaminations of physical, chemical, and microbiological nature [4]. This Special Issue focuses the attention on this last category of hazards. Microorganisms in food can have a positive, neutral, or negative role [4]. Among the possible negative impacts, we have to clearly distinguish spoilage microbes that have detrimental effects of food quality, from pathogens and producers of compounds harmful to human health, which pose risks for human health [2,4]. Hence, the focus of this collection of contributions deals with the understanding of microbiological hazards and the management of microbiological risks.

In this Special Issue on “Microbiological Safety of Foods”, we collected five original research papers that testify how broad the landscape of the different studies in the field can be. Two articles deal with pathogens diffusion and diversity in specific geographical contexts. Berthold–Pluta and co-authors [5] delved into the presence and toxic potential of *Bacillus cereus* from a particular geographical market. They assessed prevalence in about 600 samples of different Polish food products. 38.8% of the analyzed samples (both of plant and animal origin) resulted positive, leading to the selection of 1022 *B. cereus* isolates that were subjected to a tailored characterization. The authors also suggested a potential spoilage aptitude of a relevant number of isolates. Counterposed to this broad geographical study, Pažin and collaborators [6] proposed an investigation on the presence of *Listeria monocytogenes* during the production chain of fermented sausages focusing on a small-scale facility (thus, on a really circumscribed area). In this model plant, they traced the contamination routes of this emerging pathogen, providing advances in the understanding of contamination potential in a giving production unit. In particular, the authors recovered *L. monocytogenes* from the defrosting room and in association with the following phases: cutting, mixing, stuffing, and fermentation. The fact they detected the same pulsotypes in both samples from the environments of production unit and the fermented products, suggested the persistence of the pathogen in the batches of sausages, as well as in environmental samples, indicated the persistence of *L. monocytogenes* in the production plant. Remaining in the field of microbiological risks in meat, Djenane et al. [7] proposed a study on the potential in biocontrol of olive leaves extract from Algerian oleaster (*Olea europaea* var. *sylvestris*). Using raw Halal minced beef as a model food, the authors tested the impact of the extract on the microbiological safety, antioxidant properties and shelf-life stability of the product. The chemistry of the oleaster concentrate in terms of polyphenols diversity has been elucidated. The addition of the extract to raw Halal minced beef with a concentration of 5% (*v*/*w*) led to the best results, decreasing psychrotrophic counts as well as the level of *Salmonella enterica* ser. Enteritidis and Shiga toxin-producing *Escherichia coli* O157:H7, without any negative impact on the global acceptability and bitterness. Dao and co-authors [8] concentrated their attention on lettuce in urban gardens of Ouagadougou (Burkina Faso) evaluating the influence of water quality and post-harvest handling on microbiological contamination, a subject of interest for both food security and food safety in developing countries. The study showed that (i) in more than half of the samples irrigation water was not compliant with the standards of the World Health Organization (WHO), (ii) microbial contaminations augmented lengthways the trade chain, (iii) that proper post-harvest handling can be of help to avoid the proliferation of total coliforms. Finally, Bleve et al. [9] assessed the microbiological quality of jellyfish as a possible novel food in Western food markets, monitoring selected safety and quality criteria based on the standards for other European products. No *Salmonella* spp., *Listeria monocytogenes*, *Enterobacteriaceae,* and *Pseudomonas* spp. were detected, while a small occurrence of *Staphylococci* was found. The need of tailored ‘saline conditions’ was suggested to improve the performances of total bacterium, yeast and mould counts.

All five works represent subjects of interest to assure an improved management of microbial risks in food production and supply, often enhancing the sustainability of food systems. In this light, they reflect the general interest in the fields of pathogens diversity, biocontrol activities, best practices ‘from the farm to the fork’ and attention to novel foods, also looking towards more resilient food systems [5,6,7,8,9,10].
